# SPS-neutralization in tissue samples for efficacy testing of antimicrobial peptides

**DOI:** 10.1186/s12879-019-4700-1

**Published:** 2019-12-30

**Authors:** Gabrielle Sherella Dijksteel, Peter H. Nibbering, Magda M. W. Ulrich, Esther Middelkoop, Bouke K. H. L. Boekema

**Affiliations:** 1 Association of Dutch Burn Centres, Zeestraat 29, 1941 AJ Beverwijk, The Netherlands; 2 Department of Plastic, Reconstructive & Hand Surgery, Amsterdam Movement Sciences, Amsterdam University Medical Centres, Free University of Amsterdam, De Boelelaan 1117, 1081 HV Amsterdam, The Netherlands; 3 Department of Infectious Diseases, Leiden University Medical Centre, Albinusdreef 2, 2333 ZA Leiden, The Netherlands

**Keywords:** Sodium polyanethol sulfonate, Antimicrobial peptides, SAAP-148, Efficacy, Residual activity, Neutralization

## Abstract

**Background:**

Accurate determination of the efficacy of antimicrobial agents requires neutralization of residual antimicrobial activity in the samples before microbiological assessment of the number of surviving bacteria. Sodium polyanethol sulfonate (SPS) is a known neutralizer for the antimicrobial activity of aminoglycosides and polymyxins. In this study, we evaluated the ability of SPS to neutralize residual antimicrobial activity of antimicrobial peptides [SAAP-148 and pexiganan; 1% (wt/v) in PBS], antibiotics [mupirocin (Bactroban) and fusidic acid (Fucidin) in ointments; 2% (wt/wt))] and disinfectants [2% (wt/wt) silver sulfadiazine cream (SSD) and 0.5% (v/v) chlorhexidine in 70% alcohol].

**Methods:**

Homogenates of human skin models that had been exposed to various antimicrobial agents for 1 h were pipetted on top of Methicillin-resistant *Staphylococcus aureus* (MRSA) on agar plates to determine whether the antimicrobial agents display residual activity.

To determine the optimal concentration of SPS for neutralization, antimicrobial agents were mixed with PBS or increasing doses of SPS in PBS (0.05–1% wt/v) and then 10^5^ colony forming units (CFU)/mL MRSA were added. After 30 min incubation, the number of viable bacteria was assessed. Next, the in vitro efficacy of SAAP-148 against various gram-positive and gram-negative bacteria was determined using PBS or 0.05% (wt/v) SPS immediately after 30 min incubation of the mixture. Additionally, ex vivo excision wound models were inoculated with 10^5^ CFU MRSA for 1 h and exposed to SAAP-148, pexiganan, chlorhexidine or PBS for 1 h. Subsequently, samples were homogenized in PBS or 0.05% (wt/v) SPS and the number of viable bacteria was assessed.

**Results:**

All tested antimicrobials displayed residual activity in tissue samples, resulting in a lower recovery of surviving bacteria on agar. SPS concentrations at ≥0.05% (wt/v) were able to neutralize the antimicrobial activity of SAAP-148, pexiganan and chlorhexidine, but not of SSD, Bactroban and Fucidin. Finally, SPS-neutralization in in vitro and ex vivo efficacy tests of SAAP-148, pexiganan and chlorhexidine against gram-positive and gram-negative bacteria resulted in significantly higher numbers of CFU compared to control samples without SPS-neutralization.

**Conclusions:**

SPS was successfully used to neutralize residual activity of SAAP-148, pexiganan and chlorhexidine and this prevented an overestimation of their efficacy.

## Background

Efficacy is a fundamental parameter in the discovery and development of antimicrobial agents. To determine the efficacy of an antimicrobial treatment, the drug must be neutralized immediately after the treatment time to prevent an overestimation of efficacy [1]. Neutralization of the residual activity can be achieved by reducing the effective concentration of the antimicrobial agent via dilution, filtration, centrifugation, chemical inactivation and other methods [2, 3]. However, chemical inactivation is probably the most accurate procedure as the residual antimicrobials are immediately inactivated after addition of the chemicals, also known as neutralizing agents, to the test sample. Nevertheless, chemical inactivation of antimicrobial agents is not commonplace in drug efficacy testing.

Over the past years, different neutralizing agents have been used to inactivate different antimicrobials, e.g.: *i)* lecithin and polysorbate 20 have been used for the neutralization of chlorhexidine [1, 4], *ii)* sodium thiosulphate was used for iodine [5] and *iii)* chondroitin sulfate for polyhexamethylene biguanide [6]. In the absence of neutralizing agents, bacteria surviving the efficacy test may be completely eradicated by residual antimicrobial activity during sample preparation and/or microbiological quantification [7]. This shows the relevance of neutralizing agents in efficacy testing of antimicrobials.

Currently, there is an increasing interest in the development of antimicrobial peptides (AMPs) because they are highly effective against antibiotic resistant bacteria [8, 9]. AMPs eradicate bacteria by disrupting the bacterial membrane and therefore, it is believed that bacterial-resistance to AMPs is less likely to occur [10, 11]. For these reasons, AMPs are considered promising therapeutic candidates for the development of agents to combat bacterial infections not effectively responding to antibiotics. We aim to accurately determine the efficacy of highly potent AMPs using neutralizing agents.

Previously, Edberg et al. reported that aminoglycoside and polymyxin antibiotics can be neutralized selectively using sodium polyanethol sulfonate (SPS) [12]. Yet, SPS is not commonly used to neutralize residual antimicrobial activity in efficacy tests. In the current study, we investigated the efficacy of various antimicrobial agents in the presence and absence of SPS with the aim to *i)* determine the applicability of SPS for the neutralization of different antimicrobial agents and *ii)* evaluate the importance of neutralization of residual antimicrobial activity in test samples.

## Methods

### Antimicrobial agents

SAAP-148 is a synthetic AMP inspired on the structure of the human cathelicidin, LL-37 [13]. Pexiganan is an analogue of the frog peptide called magainin 2 and was previously clinically tested [14]. Both SAAP-148 and pexiganan were synthesized, purified and identified as described by Nell et al. [15]. Lyophilized peptide was dissolved in phosphate-buffered saline (PBS) (Gibco, Paisley, UK) and aliquots of the peptide in PBS were stored at − 20 °C until use. The other antimicrobial agents used in this study were 1% (wt/wt) silver sulfadiazine (SSD) cream (Pharmacy of the Medical Centre Alkmaar, Alkmaar, the Netherlands), 0.5% (v/v) chlorhexidine in 70% alcohol (Orphi Farma B.V., Lage Zwaluwe, the Netherlands), 2% (wt/wt) mupirocin in an ointment (Bactroban; GlaxoSmithKline B.V., Zeist, the Netherlands) and 2% (wt/wt) fusidic acid in an ointment (Fucidin; Leo Pharma B.V., Amsterdam, the Netherlands).

### Preparation of ex vivo models

Human skin was obtained after elective surgery at the Red Cross Hospital (Beverwijk, the Netherlands) according to institutional guidelines and following “code of conduct for responsible use”, drafted by Federa (Foundation Federation of Dutch Medical Scientific Societies). Human skin grafts with a thickness of 0.8 mm were prepared from this tissue using a dermatome (Aesculap AG & Co. KG, Tuttlingen, Germany). Excision wounds were inflicted by removing 0.3 mm of the upper part of the skin containing the epidermis using a dermatome (width 7 mm). Subsequently, the graft was cut into pieces of approximately 1 cm^2^ using a scalpel.

### Bacterial culture

Methicillin-resistant *Staphylococcus aureus* (MRSA; clinical isolates LUH14616 [16] and Mu50, ATCC 700699), *Enterococcus faecalis* (ATCC 29212), *Pseudomonas aeruginosa* (PAO1; ATCC BAA47), *Escherichia coli* (ATCC 35218) and a clinical isolate of *Acinetobacter baumannii* were used. The clinical isolate of *A. baumannii* was kindly provided by Drs. Jan Sinnige (Regional Laboratory for Medical Microbiology and Public Health Haarlem, Haarlem, the Netherlands). Bacteria were stored in Luria-Bertani (LB; Oxoid, Ltd., Basingstoke, UK) medium supplemented with 15% (v/v) glycerol at − 80 °C. LB agar plates were used to grow the inoculae at 37 °C and 5% CO_2_ overnight. To create a mid-log phase growth culture, bacteria were cultured in LB medium at 37 °C, shaken at 200 rpm. The bacterial culture was centrifuged at 3600×*g* for 5 min and the pellet was re-suspended in PBS to the desired bacterial concentration, based on the optical density at 600 nm.

### Assessment of residual antimicrobial activity

Ex vivo excision wound models were topically exposed to 20 or 200 μL of 1% (wt/v) SAAP-148 in PBS, 1% (wt/v) pexiganan in PBS, 1% (wt/wt) SSD, 0.5% (v/v) chlorhexidine in 70% alcohol, 2% (wt/wt) Bactroban, 2% (wt/wt) Fucidin or PBS for 1 h. Tissue samples were transferred to polypropylene vials containing 1 mL of PBS and a 7-mm metal bead. Tissue homogenates were prepared using a TissueLyser LT (Qiagen, Venlo, the Netherlands) set at 50 Hz for 4 min. Subsequently, 5 μL of 10-fold serially diluted 10^7^ colony forming units (CFU)/mL MRSA (LUH14616) were plated on LB agar plates and 5 μL of 10-fold serially diluted homogenates of excision wound models exposed to an antimicrobial agent or PBS were pipetted on top of the bacteria. The surviving bacteria in each dilution step were evaluated after overnight incubation of the agar plates at 37 °C and 5% CO_2_.

### SPS-neutralization of antimicrobial activity

**Fig. 1:**
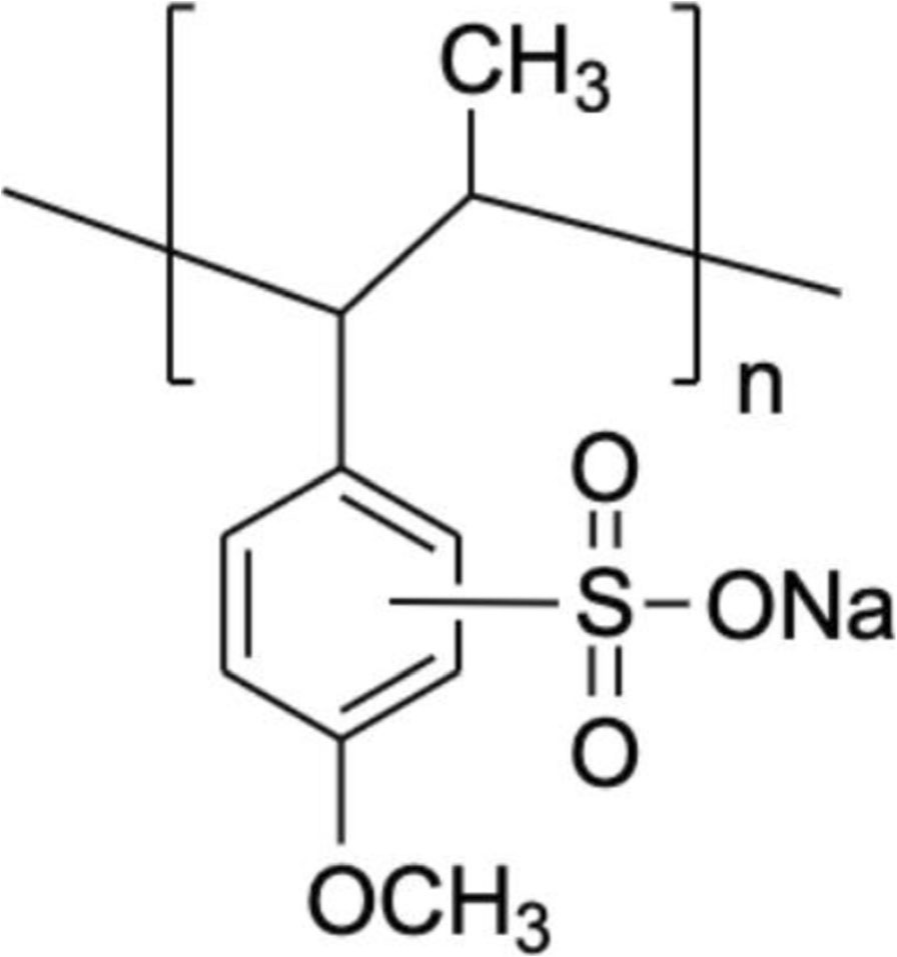
Structural formula of SPS (ChemDraw, PerkinElmer, 2018)

Ten μL of PBS or one of the antimicrobial agents: 1% (wt/v) SAAP-148 in PBS, 1% (wt/v) pexiganan in PBS, 1% (wt/wt) SSD, 0.5% (v/v) chlorhexidine in 70% alcohol, 2% (wt/wt) Bactroban or 2% (wt/wt) Fucidin were added to polypropylene vials containing 400 μL of PBS, 0.05, 0.1, 0.5% or 1% (wt/v; final concentrations) SPS (Fig. 1) in PBS (Merck, KGaA, Darmstadt, Germany). Subsequently, 90 μL of 5.6 × 10^5^ CFU/mL MRSA (LUH14616) suspension were added to the vials and the mixtures were briefly vortexed. After 30 min incubation at 37 °C and 5% CO_2_, a 7 mm metal bead was added to the vials to homogenize the samples using a TissueLyser set at 50 Hz for 4 min. This was performed to mimic the procedure of the skin samples. Ten-fold serial dilutions of the homogenates were cultured on LB agar plates to quantify the number of surviving bacteria after overnight incubation at 37 °C and 5% CO_2_.

### Efficacy testing in the absence and presence of neutralizing agent SPS

In vitro*:* Mixtures of 10 μL of 1% (wt/v) SAAP-148 in PBS or PBS and 90 μL of 10^7^ CFU/mL MRSA (LUH14616 and Mu50), *E. faecalis* (ATCC 29212)*, P. aeruginosa* (PAO1; ATCC BAA47), *E. coli* (ATCC 35218) or *A. baumannii* were incubated for 30 min at 37 °C and 5% CO_2_. Subsequently, 400 μL of PBS with or without 0.05% (wt/v) SPS and a 7-mm metal bead were added to the mixtures to prepare homogeneous suspensions using a TissueLyser set at 50 Hz for 4 min.

Ex vivo*:* Excision wound models were inoculated with 10 μL of 10^7^ CFU/mL MRSA (LUH14616) for 1 h and then topically exposed to 20 μL of 1% (wt/v) SAAP-148 in PBS, 1% (wt/v) pexiganan in PBS, 0.5% (v/v) chlorhexidine in 70% alcohol or PBS for 1 h. Thereafter, tissue samples were transferred to polypropylene vials containing a 7-mm metal bead and 1 mL of PBS with or without 0.05% (wt/v) SPS to prepare tissue homogenates using a TissueLyser set at 50 Hz for 4 min.

To determine the number of viable bacteria, 10-fold serial dilutions of the homogenates were cultured overnight at 37 °C and 5% CO_2_ on LB agar plates.

### Statistical analysis

To determine the statistically significant differences between two sample groups, the non-parametric Kruskal-Wallis test and the Mann Whitney rank-sum test were used.

## Results

### Residual antimicrobial activity in tissue samples

**Fig. 2:**
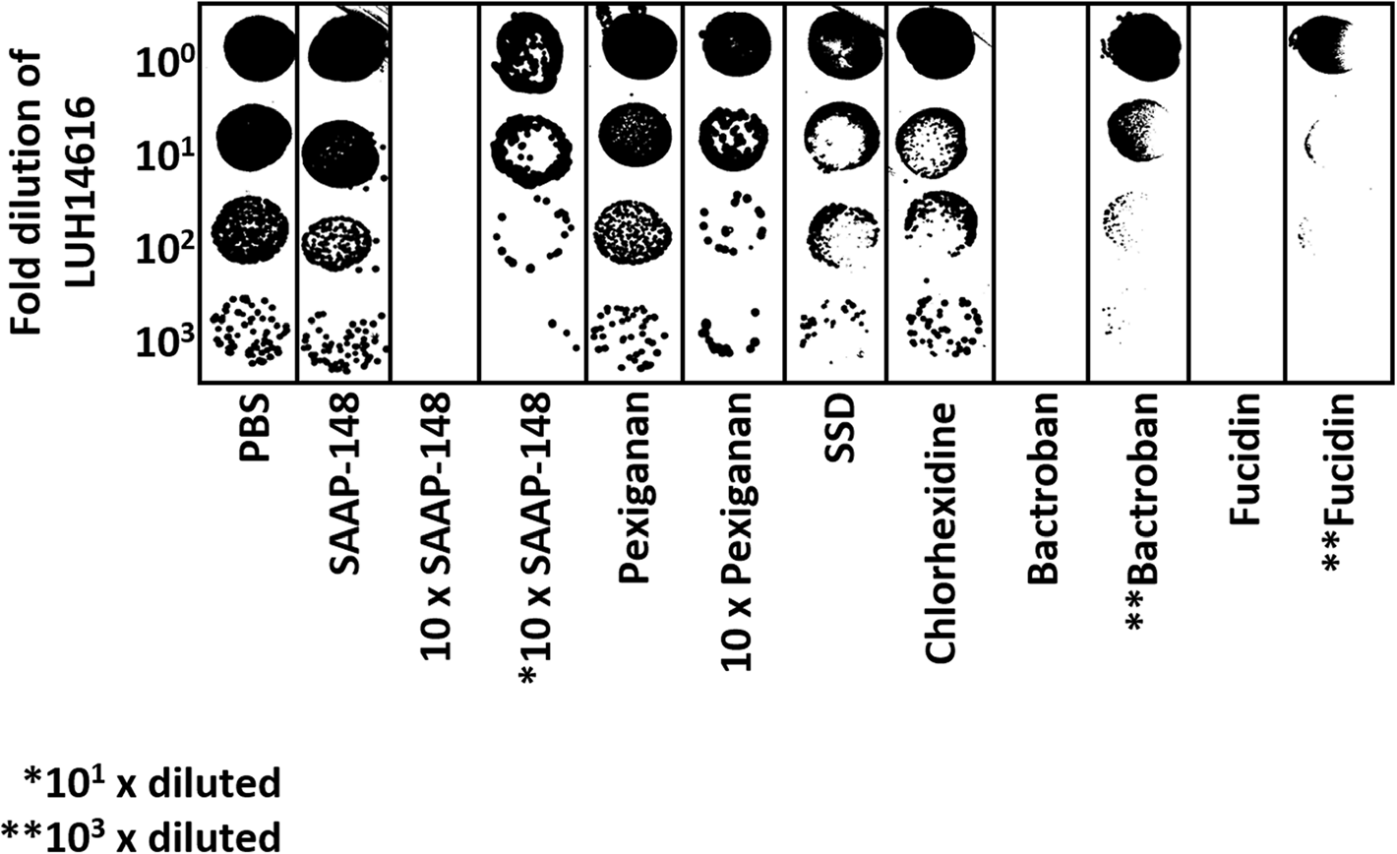
Residual antimicrobial activity. Five μL of serially diluted LUH14616 were plated on agar and 5 μL of homogenates of excision wound models exposed to 20 μL or 10-fold higher amounts (200 μL) of various antimicrobial agents or PBS were pipetted on top of the bacteria. Results of one experiment are illustrated as the surviving bacteria in each dilution step

To evaluate the presence of residual antimicrobial activity, ex vivo excision wound models were topically exposed to 20 μL of various antimicrobial agents or PBS for 1 h, and homogenates were prepared. Subsequently, serial dilutions of these homogenates were pipetted on top of serially diluted MRSA (LUH14616) suspensions on agar. All tested antimicrobial agents showed residual activity (Fig. 2). Particularly for the undiluted SSD-, chlorhexidine-, Bactroban- and Fucidin-exposed tissue homogenates bacterial killing was evident as inhibition zones appeared or bacteria were completely eradicated. Only at 1000-fold dilution of the Bactroban- and Fucidin-exposed tissue homogenates, surviving bacteria were detected. In contrast, the surviving bacteria of the SAAP-148- and pexiganan-exposed tissue homogenates were comparable to that of the PBS-exposed tissue homogenates. However, when 10-fold higher antimicrobial amounts (200 μL) were used for the SAAP-148- and pexiganan-exposed tissue homogenates, bacterial killing was observed. Inhibition zones appeared for the undiluted pexiganan-exposed tissue homogenate whereas for the undiluted SAAP-148-exposed tissue homogenate bacteria were completely eradicated. Interestingly, at 10-fold dilution of this SAAP-148-exposed tissue homogenate inhibition zones appeared (Fig. 2), indicating that at high antimicrobial concentrations residual activity is highly effective against bacteria.

### Neutralization of antimicrobial activity by SPS

**Fig. 3:**
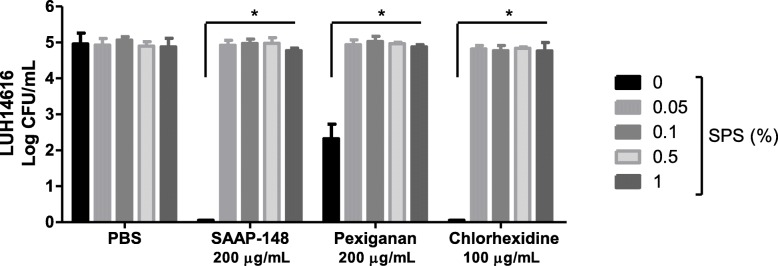
Effect of SPS on the antimicrobial activity of various antimicrobial agents. Mixtures of 10 μL of SAAP-148 (1% wt/v), pexiganan (1% wt/v), chlorhexidine (0.5% v/v in 70% alcohol) or PBS and 400 μL of PBS, 0.05, 0.1, 0.5% or 1% (wt/v; final concentrations) SPS in PBS were prepared. Ninety μL of LUH14616 with a final concentration of 10^5^ CFU/mL were added to these mixtures to determine the antimicrobial activity after 30 min incubation at 37 °C and 5% CO_2_. The means and standard deviations (SD) of three independent experiments performed in duplicate are shown. Results are expressed as the number of surviving bacteria in log10 CFU/mL. * indicates significant difference as compared to the samples without SPS (*p < 0.05)

To determine whether SPS (Fig. 1) can effectively neutralize different antimicrobial agents, mixtures containing 400 μL of PBS or 0.05, 0.1, 0.5% or 1% (wt/v) SPS in PBS and 10 μL of various antimicrobial agents or PBS were prepared. Subsequently, MRSA (LUH14616) with a final concentration of 10^5^ CFU/mL was added to these mixtures to determine the antimicrobial effect. Of note, the increasing concentrations of SPS did not affect the bacterial survival as the number of viable bacteria in the presence of SPS was comparable to the number of viable bacteria in PBS alone (Fig. 3). The antimicrobial activity of SAAP-148 (200 μg/mL), pexiganan (200 μg/mL) and chlorhexidine (100 μg/mL) was efficiently neutralized by ≥0.05% (wt/v) SPS, resulting in the complete survival of approximately 10^5^ CFU/mL MRSA (LUH14616) (Fig. 3). However, the antimicrobial activity of SSD, Bactroban, and Fucidin was not affected by these SPS concentrations as either no colonies were detected or colonies were found in 10-or more fold dilutions but not in the undiluted samples on agar (data not shown).

### Efficacy testing of antimicrobial agents in the absence or presence of SPS

**Fig. 4:**
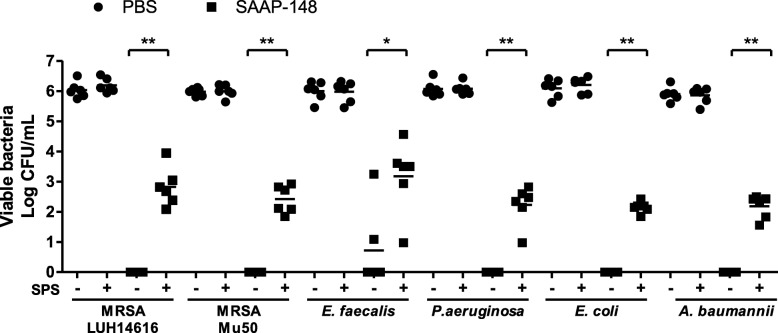
SPS-neutralization in efficacy tests of SAAP-148 against gram-positive and gram-negative bacteria. Ninety μL of 10^7^ CFU/mL MRSA (LUH14616 and Mu50), *E. faecalis* (ATCC 29212)*, P. aeruginosa* (PAO1; ATCC BAA47), *E. coli* (ATCC 35218) and *A. baumannii* were exposed to 10 μL of 1% (wt/v) SAAP-148 in PBS or PBS for 30 min. Subsequently, samples were homogenized in 500 μL of PBS with or without 0.05% (wt/v) SPS. The means and SD of six independent experiments performed in duplicate are shown. Results are expressed as the number of surviving bacteria in log10 CFU/mL. * indicates significant difference as compared to the samples without SPS (*p < 0.05); **p < 0.01)

**Fig. 5:**
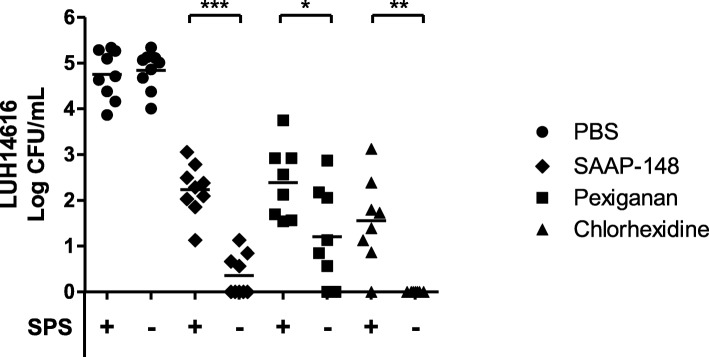
SPS-neutralization of residual activity of various antimicrobial agents. Excision wound models were inoculated with 10^5^ CFU/mL LUH14616 for 1 h and exposed to 20 μL of SAAP-148 (1% wt/v), pexiganan (1% wt/v), chlorhexidine (0.5% v/v in 70% alcohol) or PBS for 1 h. Subsequently, the models were homogenized in 1 mL of PBS with or without 0.05% (wt/v) SPS. The means and SD of at least eight independent experiments performed in triplicate are shown. Results are expressed as the number of surviving bacteria in log10 CFU/mL. * indicates significant difference as compared to the samples without SPS (*p < 0.05); **p < 0.01; ***p < 0.001)

To test the effect of 0.05% (wt/v) SPS in PBS on the efficacy of SAAP-148 against gram-positive and gram-negative bacteria, 10 μL of 1% (wt/v) SAAP-148 in PBS or PBS was mixed with 90 μL of MRSA (LUH14616 and Mu50), *E. faecalis* (ATCC 29212)*, P. aeruginosa* (PAO1; ATCC BAA47), *E. coli* (ATCC 35218) or a clinical isolate of *A. baumannii* for 30 min. Subsequently, the samples were homogenized in PBS with or without 0.05% (wt/v) SPS and the number of viable bacteria was determined. Of note, SPS did not affect the bacterial survival of these types of bacteria as the number of viable bacteria in the presence of SPS was comparable to the number of viable bacteria in PBS alone (Fig. 4). More interestingly, SPS-neutralization of SAAP-148 resulted in approximately 100 CFU/mL of surviving bacteria, whereas without SPS-neutralization, all bacteria were eradicated except for *E. faecalis* in two of six experiments (average 10 CFU/mL). The same was found for SAAP-148 (*p* < 0.001), pexiganan (*p* < 0.05) and chlorhexidine (*p* < 0.01) in ex vivo excision wound models that had been inoculated with 10^5^ CFU MRSA (LUH14616) (Fig. 5).

## Discussion

We have shown that all tested antimicrobial agents displayed residual activity in tissue homogenates, as inhibition zones appeared on the agar or the number of CFU decreased. Notably, when 20 μL of SAAP-148 (200 μg/mL) were used for the SAAP-148-exposed tissue homogenates residual activity was not evident; however, when 10-fold higher antimicrobial amounts (200 μL; 2 mg/mL) were used for the SAAP-148-exposed tissue homogenates, inhibition zones appeared when this homogenate was 10-times diluted (200 μg/mL) (Fig. 2). This suggests that SAAP-148 interacts with tissue components and that the remaining amounts of active antimicrobials were higher and thus more effective against bacteria when 10-fold higher antimicrobial amounts were used to prepare the homogenate.

As recommended in the American Society for Testing and Materials standard, we did not only determine the efficacy of the polyanionic detergent SPS (Fig. 1) in inactivating various antimicrobial agents but also determined its toxicity for the bacteria used in this study [17]. We anticipated that SPS would not neutralize the activity of SSD, Bactroban and Fucidin, due to their net negative charge at physiological conditions, which prevents the binding of SPS via electrostatic attraction. Nevertheless, SPS not only effectively neutralizes aminoglycoside and polymyxin antibiotics but also other antimicrobials, including SAAP-148, pexiganan and chlorhexidine (Fig. 3). Because SPS binds to and therefore inactivates antimicrobials depending on their cationic strength, it is believed that SPS could be more commonly used for the neutralization of AMPs in efficacy tests as they are usually highly positively charged.

Furthermore, the effect of residual activity of SSD, Bactroban and Fucidin on bacteria could be inhibited via dilution as colonies could be observed in 10-or more fold dilutions but not in the undiluted samples. However, for highly potent antimicrobials dilution of test samples may not be effective enough to eliminate the residual activity. To effectively and efficiently neutralize residual activity of antimicrobials alternatives to dilution are required. Already in 1993, Zabinski et al. reported on the use of beads that can neutralize residual activity of quinolone antibiotics [18]. We studied SPS-neutralization in efficacy tests of SAAP-148 against gram-positive and gram-negative bacteria and for different antimicrobial agents such as pexiganan and chlorhexidine. SPS-neutralization of these antimicrobials was required to prevent ongoing bacterial killing during sample preparation (Figs. 4 and 5). This is in agreement with Kampf et al., who reported that neutralizing agents were required to effectively inactivate a chlorhexidine-containing hand rub in efficacy tests [1, 4]. Thus, accurate neutralization of residual antimicrobial activity in efficacy tests can prevent an overestimation of the drug’s efficacy.

Previously, MacDonald et al. reported a minimal inhibitory concentration of ≤16 μg/mL for pexiganan against bacteria isolated from infected diabetic foot ulcers. This in vitro efficacy of pexiganan was not superior but equivalent to the conventional antibiotic ofloxacin [19]. A higher dosage of 2% (20,000 μg/mL) pexiganan would be more favorable in the clinical studies for the treatment of infected diabetic foot ulcers. However, pexiganan failed to demonstrate its superiority over ofloxacin with statistically significant data in the phase III clinical trials [14]. As no neutralizing agents were used in these studies, the efficacy of pexiganan might be overestimated, especially because a high antimicrobial dosage was used in vivo. In line with this suggestion, we emphasize the importance of neutralization of residual antimicrobial activity in efficacy testing of novel AMPs, such as SAAP-148.

## Conclusions

Depending on the antimicrobial agent and the concentration used, residual activity in tissue samples can be high. Residual activity of different antimicrobial agents, including SAAP-148, pexiganan and chlorhexidine, can be neutralized using SPS. As a consequence, an overestimation of the drug’s efficacy is prevented. Thus, accurate preclinical efficacy testing of novel AMPs, using SPS-neutralization, will allow appropriate designs for clinical testing, if relevant.

## Data Availability

The datasets used and analyzed during the current study are available from the corresponding author on reasonable request.

## Abbreviations

AMP: Antimicrobial peptide
CFU: Colony forming units
LB: Luria-Bertani
MRSA: Methicillin-resistant *Staphylococcus aureus*
PBS: Phosphate-buffered saline
SD: Standard deviations
SPS: Sodium polyanethol sulfonate
SSD: Silver sulfadiazine
